# *De novo* assembly of transcriptomes, mining, and development of novel EST-SSR markers in *Curcuma alismatifolia* (Zingiberaceae family) through Illumina sequencing

**DOI:** 10.1038/s41598-019-39944-2

**Published:** 2019-02-28

**Authors:** Sima Taheri, Thohirah Lee Abdullah, M. Y. Rafii, Jennifer Ann Harikrishna, Stefaan P. O. Werbrouck, Chee How Teo, Mahbod Sahebi, Parisa Azizi

**Affiliations:** 1 Department of Crop Science, Faculty of Agriculture, Universiti Putra Malaysia, 43400 Serdang, Selangor Malaysia; 2 Laboratory of Climate-Smart Food Crop Production, Institute of Tropical Agriculture and Food Security, Universiti Putra Malaysia, 43400 Serdang, Selangor Malaysia; 3 Institute of Biological Sciences, Faculty of Science, University of Malaya, 50603 Kuala Lumpur, Malaysia; 4 Centre of Research in Biotechnology for Agriculture (CEBAR), University of Malaya, 50603 Kuala Lumpur, Malaysia; 5 Laboratory of Applied Science In Vitro Plant Biotechnology, Department of Plants and Crops, Faculty of Bioscience Engineering, University Ghent, Valentin Vaerwyckweg 1, BE-9000 Gent, Belgium

**Keywords:** Agricultural genetics, Molecular engineering in plants, Plant breeding

## Abstract

*Curcuma alismatifolia* widely used as an ornamental plant in Thailand and Cambodia. This species of herbaceous perennial from the Zingiberaceae family, includes cultivars with a wide range of colours and long postharvest life, and is used as an ornamental cut flower, as a potted plant, and in exterior landscapes. For further genetic improvement, however, little genomic information and no specific molecular markers are available. The present study used Illumina sequencing and *de novo* transcriptome assembly of two *C*. *alismatifolia* cvs, ‘Chiang Mai Pink’ and ‘UB Snow 701’, to develop simple sequence repeat markers for genetic diversity studies. After *de novo* assembly, 62,105 unigenes were generated and 48,813 (78.60%) showed significant similarities versus six functional protein databases. In addition, 9,351 expressed sequence tag-simple sequence repeats (EST-SSRs) were identified with a distribution frequency of 12.5% total unigenes. Out of 8,955 designed EST-SSR primers, 150 primers were selected for the development of potential molecular markers. Among these markers, 17 EST-SSR markers presented a moderate level of genetic diversity among three *C*. *alismatifolia* cultivars, one hybrid, three *Curcuma*, and two *Zingiber* species. Three different genetic groups within these species were revealed using EST-SSR markers, indicating that the markers developed in this study can be effectively applied to the population genetic analysis of *Curcuma* and *Zingiber* species. This report describes the first analysis of transcriptome data of an important ornamental ginger cultivars, also provides a valuable resource for gene discovery and marker development in the genus *Curcuma*.

## Introduction

**Figure 1:**
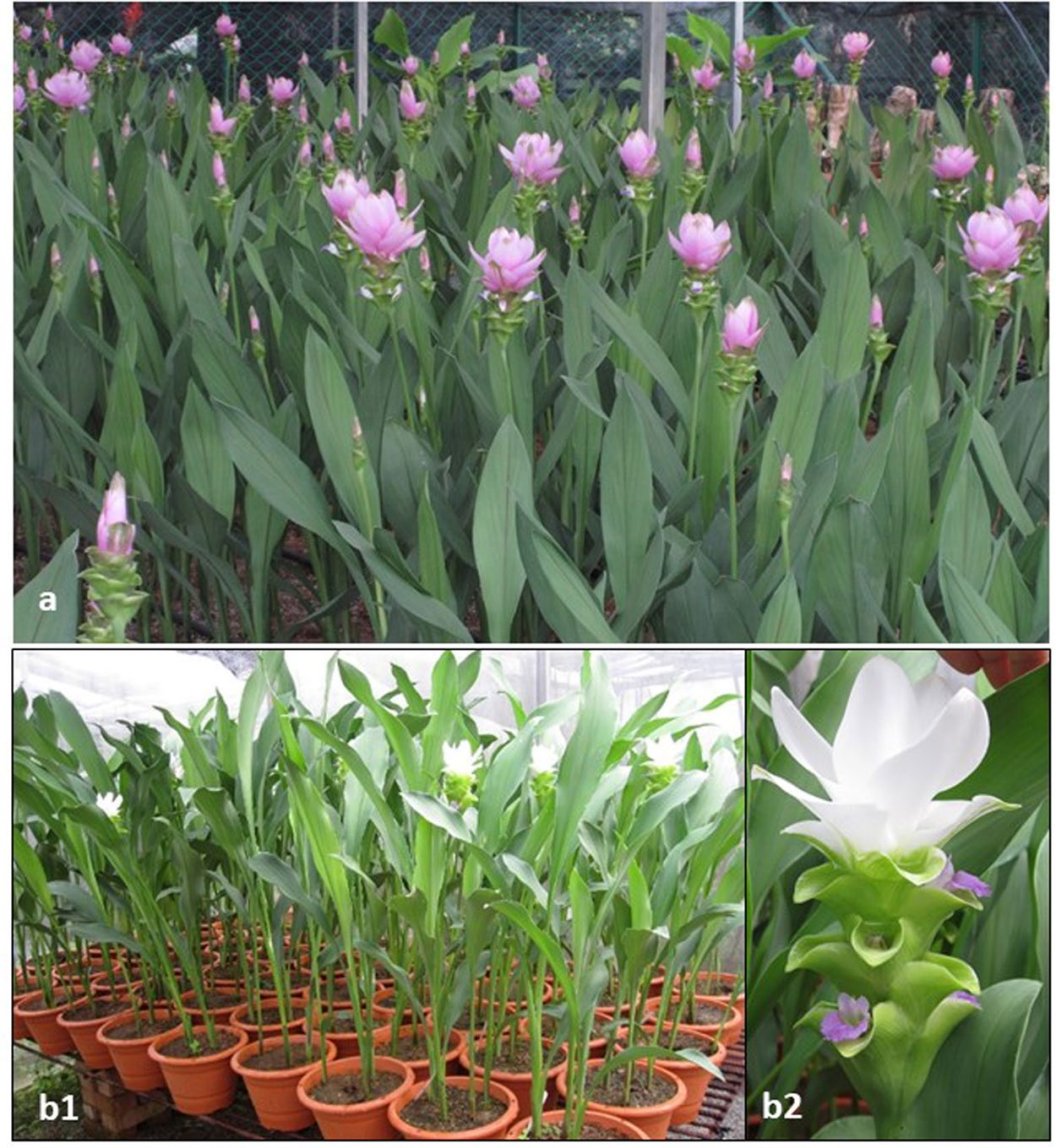
*Curcuma alismatifolia* cultivars. (**a**) Chiang Mai Pink; (**b1**) UB Snow 701; (**b2**) UB Snow 701 inflorescence.

As a member of the Zingiberaceae family, the genus *Curcuma* has only recently become popular as an ornamental. Previously, most of its species were used either for culinary purposes or as medicinal herbs. However, *Curcuma alismatifolia* Gagnep. has remarkable aesthetic value. This species of perennial originating from tropical and subtropical areas of Cambodia and northern Thailand shows great potential for use as a garden plant in tropical landscaping within various regions and as flowering potted plants, as well as cut flowers. The flowering stem consists of a showy inflorescence with several apical bracts on a long peduncle. The purplish pink/white distal bracts are more numerous than the basal green bracts. These two types of bracts come into two to seven small auxiliary flower buds. Open flowers are small with purple flag petals (Fig. 1). To improve this species, traditional breeding programmes have been launched in previous studies^1^. A large number of cultivars and hybrids with variation in flower colour, from dark purple to white, are now available. Most commercially important cultivars of *C*. *alismatifolia* are hybrids and are propagated vegetatively.

To estimate genetic variation with high reproducibility within a variety of plant species, SSRs or microsatellites are known to be highly effective tools and are considered a robust class of molecular markers^2^. SSRs are classified based on their source (i.e., genomic SSRs or g-SSRs and genic SSRs or expressed sequence tags SSRs as EST-SSRs) within the coding region and are identified from transcribed RNA sequences^3^. Although EST-SSRs are closely linked to functional genes with a possible impact on important agronomic characters, g-SSRs are not necessarily expected to be either strongly linked with transcribed regions of the genome or to have genetic functions. A major disadvantage associated with EST-SSRs is sequence redundancy that results in multiple sets of markers at the same locus. This issue can be addressed through assembling short reads of RNA transcripts and ESTs into unigenes^4,5^. Before next generation sequencing (NGS) technologies, the development of SSR markers was costly and exhibited low throughput due to the necessity of building up genomic libraries for targeted SSR motifs to create recombinant DNA molecules using restriction enzymes for DNA fragmentation. Additionally, cloning of DNA fragments into a vector was performed, as well as sequencing of clones carrying SSRs^6^. Application of molecular markers to study genetic variation of *Curcuma* species has been reported in previous studies^7–12^. Moreover, in the Zingibaraceae family, within spice crops, such as turmeric (*Curcuma longa* L.)^10,13–16^, *Zingiber officinale*^17,18^, and cardamom (*Elettaria cardamomum* Maton)^19^ genomic SSRs and EST-SSRs have been developed. The development of SSRs based on transcriptome data can therefore be viewed as useful for estimation of genetic diversity and population structure to support strategies for the breeding of populations of *C*. *alismatifolia*.

The advent of NGS technologies, such as sequencing by synthesis (e.g., Illumina) can simplify the creation of an enormous amount of genomic or transcriptome sequence data, allowing cost-effective and efficient development of molecular markers, including SSR markers^20^.

RNA sequencing (RNA-seq) is a powerful technique because the dual aspects of quantification and discovery are blended within a single sequencing assay. It is possible to easily provide information on transcriptomes through the technology of high-throughput mRNA sequencing, which is regarded as a cost-effective and powerful tool for profiling gene expression within non-model organisms that have no reference genome^21^. RNA-seq technology and *de novo* transcriptome assembly are considered effective approaches to detect functional genes, as well as to characterize patterns of gene expression and associated regulatory networks^22–31^, having mapped out a direct, reliable, and simple strategy for cost-effective and efficient development of high-throughput identification of EST-SSRs^4^. Using RNA-seq technology, EST-SSRs have been utilized and developed in a variety of plant species, such as peanut^32^, bean^33^, Chinese cabbage^34^, *Petunia* spp.^35^ mango^36^, Chinese bayberry^37^, and *Tapiscia sinensis*^38^. A list of plant species in which genomic and EST-SSRs have been developed using 454 and Illumina sequencing platforms is available in our review article^2^. Assembly of *de novo* transcriptomes have also been performed in *Curcuma longa* L., revealing novel transcripts related to antimalarial terpenoids and anticancer within rhizomes^39^ and *Zingiber officinale* cv. Suruchi of Odisha^40^. To the best of our knowledge, however, there are no previous reports of *de novo* transcriptome assembly (RNA-seq) for *C*. *alismatifolia*, particularly for the development of EST-SSR markers. In this study, we present the first *de novo* transcriptome analysis of ornamental *Curcuma* species, *C*. *alismatifolia* cv. ‘Chiang Mai Pink’ and cv. ‘UB Snow 701’ based on Illumina HiSeq 4000 sequencing and the identification of SSR markers for *Curcuma* species. This transcriptome analysis offers new insights into the evolutionary origin of *Curcuma*, as well as a rich resource for genetic information that could be used for genetic improvement and breeding.

## Results

### Illumina sequencing and *de novo* assembly of paired-end reads

In the present study, approximately 13.19 Gb of raw read data was generated from Illumina HiSeq sequencing of the upper bracts of *C*. *alismatifolia* cultivars, ‘Chiang Mai Pink’ and ‘UB Snow 701’. After raw read filtering, clean reads were assembled into 46,829 and 52,788 unigenes, reaching a total length of 40,992,261 bp and 50,231,516 bp for ‘Chiang Mai Pink’ and ‘UB-Snow 701’ cultivars, respectively. The assembled unigene length ranged from 301 to over 3000 bp with an average of 875 and 951 bp and N50 of 1,338 and 1,445 bp for ‘Chiang Mai Pink’ and ‘UB-Snow 701’ cultivars, respectively (Table 1).

**Table 1:** Summary of assembly results for ‘Chiang Mai Pink’ (CMP) and UB Snow 701’ (UBS).

Features	CMP	UBS	All-unigenes
Total Raw Reads (Mb)	69.97	69.97	
Total Clean Reads (Mb)	65.82	66.11	
Total Clean Bases (Gb)	6.58	6.61	
Clean Reads Q20 (%)	99.06	98.94	
Clean Reads Ratio (%)	94.07	94.48	
Total Number of transcripts	65,539	80,206	
Total Length of transcripts (bp)	50,262409	64,588299	
Mean Length of transcripts (bp)	766	805	
N50 value of transcripts	1250	1345	
GC(%)	47.27	47.22	
Total number of unigenes	46,829	52,788	62,105
Total length of unigenes (bp)	40,992,261	50,231,516	61,786,426
Mean length unigenes (bp)	875	951	994
N50 value of unigenes	1,338	1,445	1,501
GC(%)	47.32	47.26	47.23

Q20: The rate of bases which quality is greater than 20; N50: a weighted median statistic that 50% of the total length is contained in unigenes great than or equal to this value. GC (%): the percentage of G and C bases in all unigenes.

**Figure 2:**
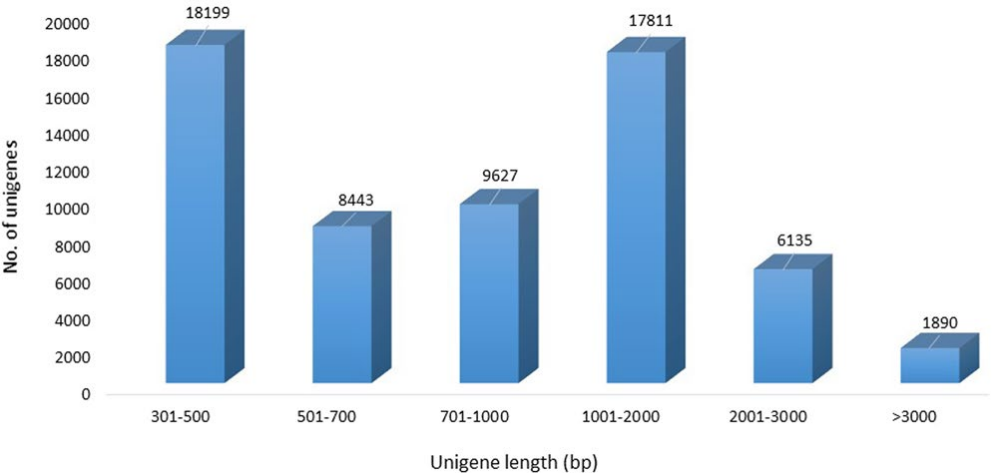
Length distribution of the assembled unigenes in *C*. *alismatifolia*.

Of all unigenes (62,105), 58.4% (36,269) were shorter than 1,000 bp, 38.5% (23,946) of unigenes ranged from 1,000 to 3,000 bp, and only 3.04% (1,890) of unigenes were longer than 3,000 bp (Fig. 2). In *C*. *alismatifolia*, these unigenes offer a potential source for the identification of functional molecular markers and genes.

### Functional annotation and classification of the unigenes

**Figure 3:**
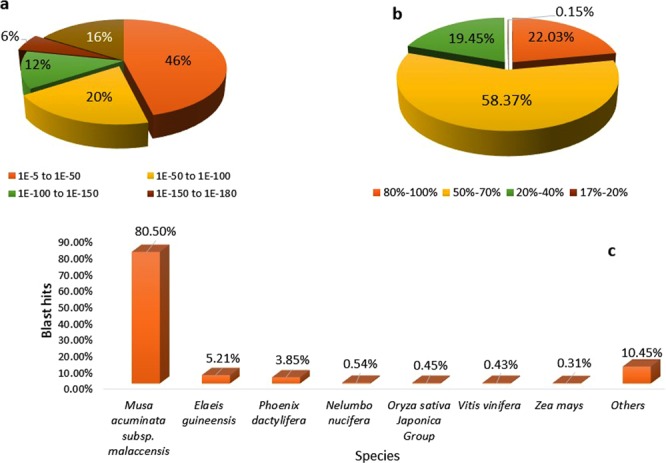
Characteristics of homology analysis for *C*. *alismatifolia* unigenes against the non- redundant protein database (Nr) with an E-value of 10^−5^, (**a**) The E-value distribution of BLASTx hits for each assembled unigene, (**b**) The similarity distribution of BLASTx hits for each assembled unigenes, (**c**) Species-based distribution of the top BLASTx hits for each assembled unigenes.

**Figure 4:**
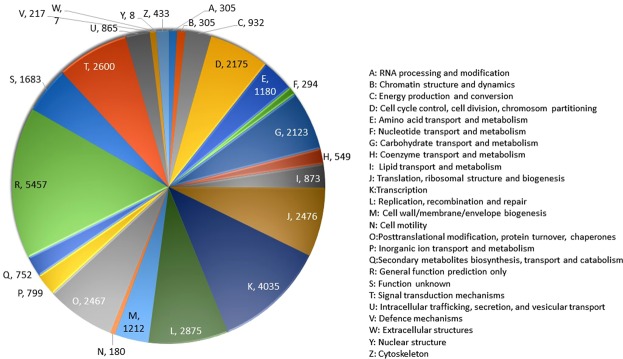
Distribution of Cluster of Orthologous Group (COG) classification. A total of 19,546 assembled unigenes were annotated and assigned to 25 functional categories. Subgroups in the COG classification by colour and the number of genes in each main category are identified on the chart.

After sequence contig assembly, unigenes were annotated by comparison against the six functional databases: NCBI Nr and Nt; COG; GO; KEGG and Swiss-Prot (Table 2, Supplementary Table S1). As indicated in Table 1, 47,160 (75.94%) of unigenes were significantly matched with known proteins in the Nr database, while 44,007 (70.86%) unigenes matched with entries in the nucleotide sequence (Nt) database, and 33,298 (53.62%) matched with proteins in the Swiss-Prot database. To further analyse BLAST results, *E*-value and similarity distributions were calculated using the Nr database. The *E*-value distribution of the top hits revealed that 53.98% of annotated sequences had high scores for homology (*E*-value < 10^−50^), whereas 46.02% showed homology with *E*-values ranging from 10^−5^ to 10^−50^ (Fig. 3a). Additionally, 80.4% of sequences were found to have similarities of over 70% (Fig. 3b). These results reflect the high identities of the mapped sequences with known sequences, suggesting good assembly quality. Species distribution showed *Musa acuminata* subsp. *malaccensis* ‘DH Pahang’ (Musaceae) (order Zingiberales) to have a very high similarity score with 37,962 (80.5%) top BLASTx hits. Other species matched at below 6%, including African oil palm, *Elaeis guineensis* (Arecaceae) with 2,456 (5.21%) and date palm, *Phoenix dactylifera* (Arecaceae) with 1,815 (3.85%) (Fig. 3c). Further functional prediction and classification of all unigenes was performed using their annotation with COG, GO, and KEGG databases (Table 2, Supplementary Table S1). COG function classification of the sequence of *C*. *alismatifolia* produced Nr hits for19,546 of 62,105 unigenes which, were annotated and classified functionally into 25 COG functional categories, including biochemistry metabolism, cellular structure, signal transduction, and molecular processing (Fig. 4). The cluster for general function prediction represented the largest group with 5,457 genes (15.6%) followed by transcription (4,035, 11.6%) and replication, recombination and repair with 2,875 genes (8.26%). Additionally, only a few unigenes were assigned to nuclear structure and extracellular structures with eight (0.02%) and seven (0.02%) genes, respectively.

**Table 2:** Summary of functional annotation of unigenes of *C*. *alismatifolia* with six databases.

Values	Total unigenes	Nr-Annotated	Nt-Annotated	Swissprot-Annotated	KEGG-Annotated	COG-Annotated	GO-Annotated	Overall*
Number	62,105	47,160	44,007	33,298	35,629	19,546	3,651	48,813
Percentage	100%	75.94%	70.86%	53.62%	57.37%	31.47%	5.88%	78.60%

*Overall: the number of unigenes which be annotated with at least one functional database.

**Figure 5:**
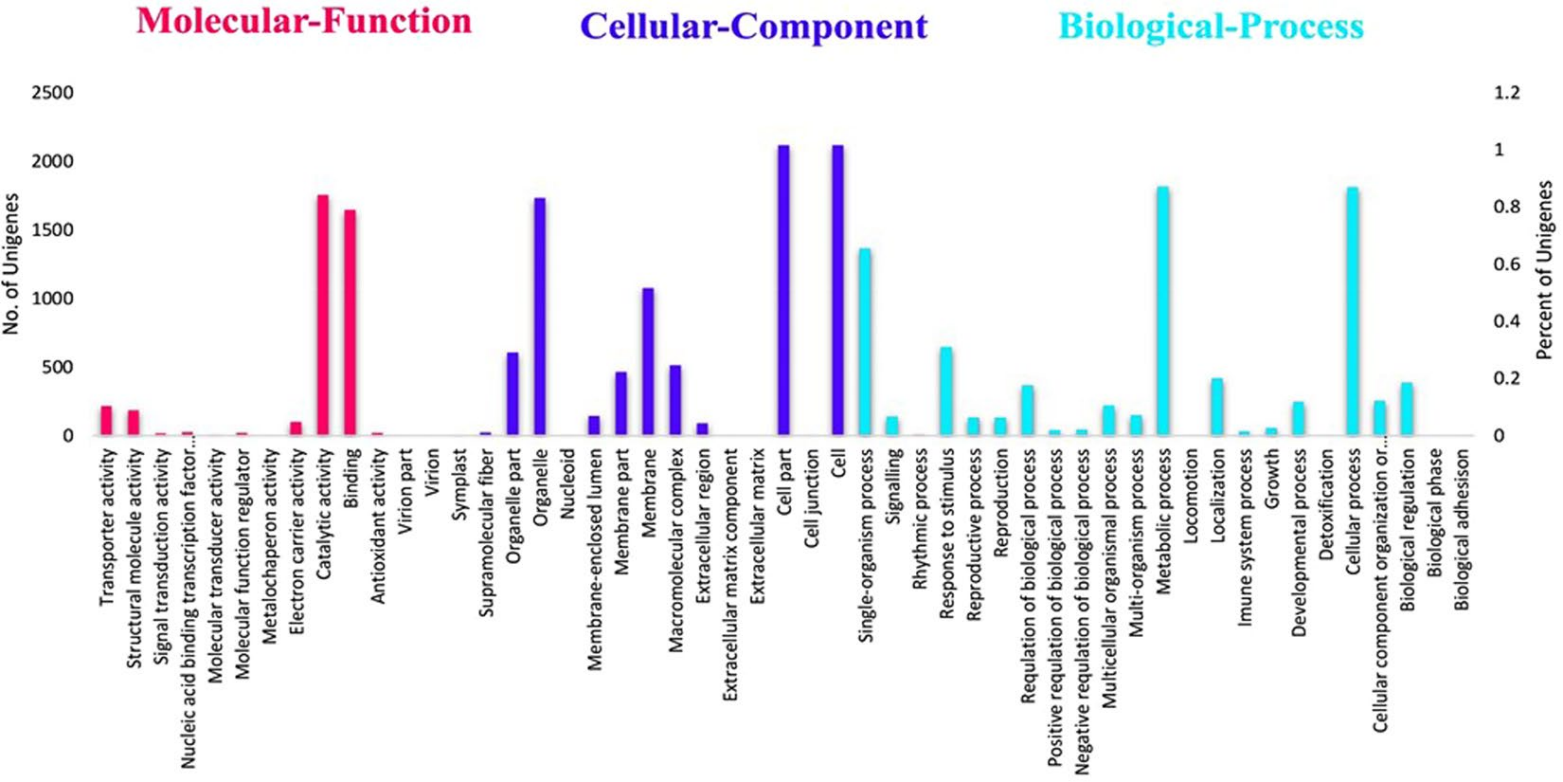
Gene ontology (GO) classification of assembled unigenes of *C*. *alismatifolia*. Results are summarized in three main categories: molecular function, cellular component, biological process. The x-axis indicates the subgroups in GO annotation while the y-axis indicates the percentage of specific categories of genes in each main category.

In the present study, 3,651 (5.88%) *C*. *alismatifolia* unigenes were assigned to GO classes based on Nr annotation with 21,210 functional terms (Fig. 5). The annotated unigenes that belonged to three clusters of molecular function, cellular component, and biological process were categorized into 51 functional groups. Cellular component (8,917, 42.04%) and biological process (8,292, 39.09%) classifications represented the largest number of unigenes followed by molecular function (4001, 18.87%). Under the cellular component category, three subcategories, cell, organelle, and membrane, represented 38.28% of associated unigenes. The majority of the unigenes in the biological process category were specific for metabolic (1,817, 8.56%) and cellular (1,814, 8.55%) processes followed by single-organism processes (1,367, 6.44%) and stimulus response (648, 3.05).

**Figure 6:**
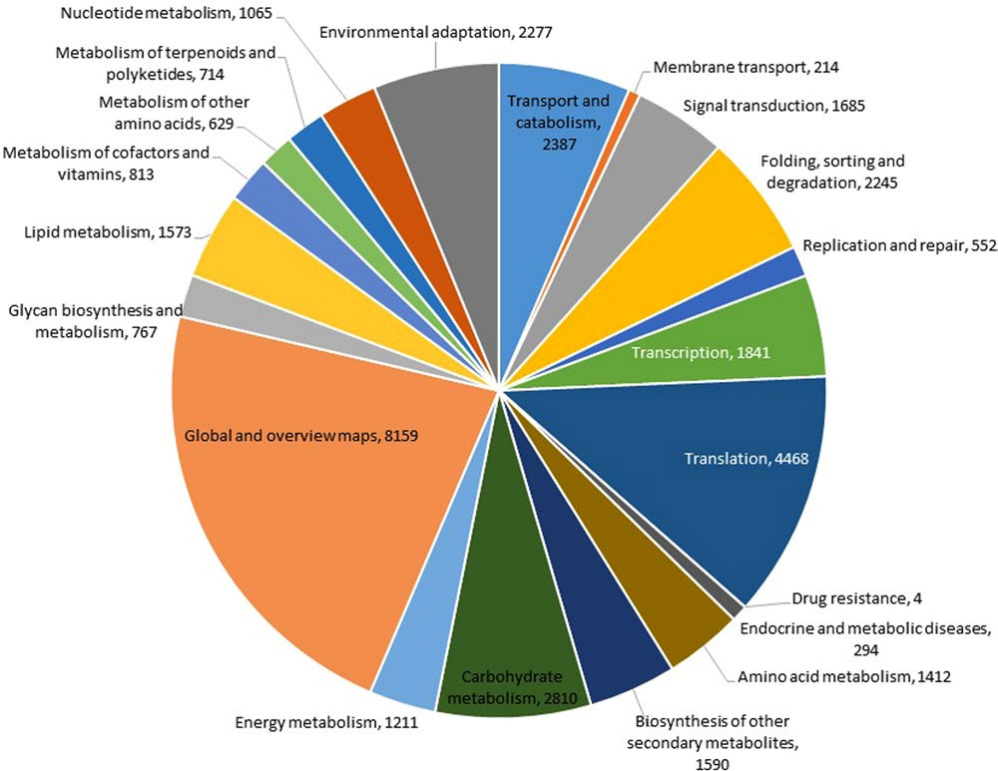
The top 21 KEGG metabolic pathways of assembled unigenes. The number of genes in each metabolic pathway are indicated.

All unigenes were analysed by comparison with the KEGG pathway database for further analysis of the *C*. *alismatifolia* transcriptome. Carrying significant matches in this present study, 35,629 (57.37%) unigenes were assigned to 135 predicted metabolic pathways. Figure 6 shows the top 21 pathways based on six main categories. The metabolism pathway was the most represented among these six main categories, containing 20,743 unigenes (56.50%), followed by genetic information processing (9,106, 24.80%), cellular processes (2,387, 6.50%), organismal systems (2,277, 6.20%), environmental information processing (1,899, 5.17%), and human diseases pathways (298, 0.81%). These functional annotations provide useful information to further investigate specific developmental and biochemical processes of *C*. *alismatifolia*, as well as potential functions, structures, and pathways of genes.

### Identification of EST-SSRs from *C*. *alismatifolia* transcriptome

**Figure 7:**
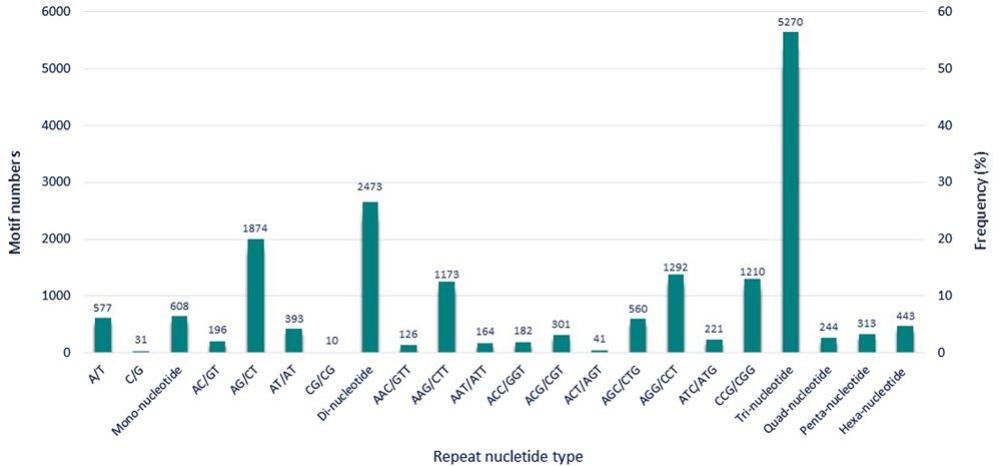
Frequency distribution of SSRs based on motif sequence type and number.

Using MISA software, all 62,105 unigenes were employed to mine potential EST-SSRs for development of SSRs in *C*. *alismatifolia*. A total of 7,811 unigene sequences were found to encode 9,351 potential EST-SSRs (Supplementary Table S2). Of these, 1,248 unigenes contained more than one SSR and 501 SSRs were present in compound formation (Table 3). Table 4 and Fig. 7 offer a summary of the number and type of EST-SSRs along with different numbers of tandem repeats. From 61,786.426 kb of examined sequences, we detected a frequency of at least one SSR per 6.6 kb within the *C*. *alismatifolia* genome expressed fraction. In identified SSR loci considering sequence complementary, 213 motif sequence types were identified, of which mono, di-, tri-, quad-, penta-, and hexa- nucleotide repeats had 2, 4, 10, 26, 57, and 114 types, respectively (Fig. 7). Trinucleotide repeat motifs were the most abundant (5,720, 56.35%) followed by dinucleotide repeat motifs (2, 473, 26.44%), whereas hexa- (443, 4.74%), penta- (313, 3.35%) and quad- nucleotide repeat motifs (244, 2.61%) were rare. Within the two possible types of mononucleotide repeat, A/T was the most abundant motif, containing 6.17% of total SSRs. The C/G motif was less abundant than the A/T with frequency of 0.33%. Among the dinucleotide repeat motif types, AG/CT, with frequency of 20.04% was the most abundant repeat motif while CG/CG was the least abundant motif, constituting 0.1% of total SSRs. The two most frequent repeats among the trinucleotide repeat motifs were AGG/CCT (13.82% of total SSRs) and CCG/CGG (12.94% of total SSRs) followed by AAG/CTT (12.5%). Quad-, penta-, and hexa- nucleotide repeats motifs constituted 10.70% of the total SSRs (Table 4, Fig. 7). The number of SSR repeats ranged from 4 to 29, with five repeats (2,983, 31.90%) representing the most common followed by six (2,335, 24.97%) and seven (1,211, 12.95%) tandem repeats.

**Table 3:** Summary of EST-SSRs identified from *C*. *alismatifolia*.

Features	
Total number of sequences examined 62,105	62,105
Total size of examined sequences (bp) 61,786,426	61,786,426
Total number of identified SSRs 9,351	9,351
Number of SSR containing sequences 7,811	7,811
Number of sequences containing more than one SSR 1,248	1,248
Number of SSRs present in compound formation 501	501

**Table 4:** Summary of EST-SSRs identified from the unigenes of *C*. *alismatifolia*.

Repeat motifs	No. of repeats									
	4	5	6	7	8	9	10	>10	Total	Frequency (%)
**Mono-nucleotide**										
A/T	—	—	—	—	—	—	—	577	577	6.17
C/G	—	—	—	—	—	—	—	31	31	0.33
									608	6.5
**Di**-**nucleotide**										
AG/CT	—	—	572	368	264	212	129	329	1,874	20.04
AT/AT	—	—	150	99	53	30	19	42	393	4.2
AC/GT	—	—	90	41	32	13	9	11	196	2.09
CG/CG	—	—	7	1	1	1	—	—	10	0.1
									2,473	26.44
**Tri**-**nucleotide**										
CCG/CGG	—	655	320	142	91	—	1	1	1,210	12.94
AGG/CCT	—	603	375	180	133	—	1	—	1,292	13.82
AAG/CTT	—	542	296	200	135	—	—	—	1,173	12.54
AGC/CTG	—	322	150	53	34	1	—	—	560	5.99
ACG/CGT	—	177	69	41	11	—	1	2	301	3.21
Others	—	420	190	78	38	4	4	—	734	7.85
									5,270	56.35
**Quad**—**nucleotide**										
AAAG/CTTT	—	21	11	—	1	—	—	—	33	0.35
AAAT/ATTT	—	20	5	—	—	—	—	—	25	0.26
AAGG/CCTT	—	16	14	—	—	1	—	—	31	0.33
AAAC/GTTT	—	13	9	—	—	—	—	—	22	0.23
Others	—	86	42	3	—	1	—	1	133	1.42
									244	2.61
Penta-nucleotide	237	63	12	1	—	—	—	—	313	3.35
Hexa-nucleotide	369	45	23	4	—	2	—	—	443	4.74
Total	606	2,983	2,335	1,211	793	265	164	994	9,351	100
Frequency (%)	6.48	31.9	24.97	12.95	8.48	2.84	1.75	10.63	100

### SSR primer design and validation

Out of 9,351 SSRs, 8,955 high-quality primer pairs were successfully designed, and 150 primers were randomly selected for amplification of genomic DNA from *C*. *alismatifolia* (three cultivars and one hybrid), three *Curcuma* species, and two *Zingiber* species. Of the 150 tested primers, 70 primers amplified reproducible amplicons with the expected band sizes. Considering the amplification and polymorphic loci, 17 SSR primer pairs that presented clear and polymorphic loci were selected to evaluate polymorphism levels within the *Curcuma* and *Zingiber* species (Table 5, Supplementary File S3). Characteristics associated with the 17 polymorphic EST-SSR markers are listed in Table 6. The number of alleles (Na) per marker ranged from 3 to 6, with 75 alleles in total. The maximum and minimum levels of effective number (Ne) of alleles per locus was 4.378 and 1.975 with an average of 3.24. Observed heterozygosity (Ho) varied from 0 to 0.555 with an average of 0.163, whereas expected heterozygosity (He) varied from 0.499 to 0.780 with an average of 0.685. With a mean value of 1.285, the Shannon index (I) ranged from 0.848 to 1.642. The value of polymorphic information content (PIC) ranged from 0.437 to 0.743 with a mean value of 0.627.

**Table 5:** Characteristics of primers designed for analyzing genetic diversity of *C*. *alismatifolia*.

Primer ID	SSR motif	Primer pair	Tm (°C)	Product size (bp)
CuAl04	GCC(3*6)	F: GAGAGATCAGTCATCCCTATTCG R: ACAACGTTATTATTGCCTGGAGA	59.2	100–125
			59.9	
CuAl08	GGT(3*6)	F: CAGACACTTATCGTCGTTGGTTA R: AAACTGAAACATACTCCACCACC	59.2	100–165
			59.3	
CuAl10	GCA(3*5)	F: TCTTCTGCTAGATACTTCAGCGG R: TGTCTGGGGAAATCACTAACATC	60.1	120–140
			60.2	
CuAl11	AAT(3*5)	F: CATTATCTGTTCACTGGTAGCCC R: AAATTTGAACTGTTTCCTGATGC	59.9	130–155
			59.5	
CuAl12	CCGGCT(6*4)	F: CACATCGGAAATTTAAGCATCA R: GGCTCCTGAACCACCACC	59.9	145–200
			61.5	
CuAl13	GAA(3*6)	F: AAAGAAGGCCTCTTCATCATCTC R: AAGCCATCTTTCTCCTTCTTCCT	60.2	85–100
			61.0	
CuAl14	TCGA(4*5)	F: CTTGACTCCATCTCTCCATTCAG R: AGTGTTCCACTACGGGGACTAAT	60.2	100–135
			60.1	
CuAl15	CTG(3*8)	F: CTTGACGAGATTCGAGGTGAC R: GCTGGCTTACTACATGGATTCTG	59.8	95–125
			60.1	
CuAl16	CGAT(4*5)	F: TTGCTTCTTTCGTCTCTTGATTC R: AACAGTGAGATCGATCCAGGTAA	60.0	125–155
			60.0	
CuAl17	TGG(3*5)	F: GCTATTCTTCAGCAAAACAAAGG R: TTGGAGCACAAAATAACAACTCA	59.4	115–140
			59.6	
CuAl18	GGT(3*5)	F: CAGAAACGACAAGGCTCTGAC R: GTAGAGCAGAGTTATGGAGTCGC	60.0	120–155
			59.5	
CuAl20	GAG(3*8)	F: AAGACGTATGTCTCCGAGATGC R: GTGAAACAGAGGAGAAGAACGAC	60.6	115–140
			59.4	
CuAl21	TCC(3*6)	F: CAATTCATCCCTCGTCAGAGT R: CGGCTAGGGAGTTGGGAT	59.1	100–140
			60.0	
CuAl22	CTG(3*5)	F: GTGCTTCATCCTCTGGGACTT R: CATCTTAGCTCTACCCAAATCCA	60.6	130–205
			59.6	
CuAl23	TCCTTC(6*5)	F: CTCCTCCACCTCACTAATTTCCT R: AGGAGTACCTCATGAAGAGCCA	60.0	150–185
			60.2	
CuAl24	TTGCT(5*5)	F: ACGTTCAAGATTTCCGAGGATAC R: GATAAATCAACCCAAATGGACAA	60.7	115–130
			59.9	
CuAl25	CTTC(4*5)	F: GACTTCTGTGCTGACAAGTCAAA R: CTTCTTTTCTCCGCAATTAGGAT	59.5	155–205
			60.1

**Table 6:** Characteristics of the 17 polymorphic EST-SSR markers in nine *Curcuma* and *Zingiber* species.

No.	Primer ID	Na	Ne	Ho	He	I	PIC
1	CuAl04	6	4.378	0.111	0.780	1.642	0.743
2	CuAl08	5	3.176	0.111	0.692	1.377	0.649
3	CuAl10	5	4.378	0.222	0.780	1.534	0.734
4	CuAl11	4	2.347	0.222	0.580	1.013	0.500
5	CuAl12	4	3.521	0.000	0.724	1.310	0.662
6	CuAl13	3	2.793	0.000	0.649	1.060	0.567
7	CuAl14	5	2.655	0.444	0.630	1.226	0.579
8	CuAl15	5	3.306	0.555	0.705	1.365	0.651
9	CuAl16	5	3.521	0.000	0.724	1.427	0.677
10	CuAl17	3	2.655	0.222	0.630	1.026	0.544
11	CuAl18	4	3.306	0.111	0.705	1.276	0.642
12	CuAl20	4	2.945	0.222	0.667	1.223	0.611
13	CuAl21	3	2.793	0.000	0.649	1.060	0.567
14	CuAl22	5	4.263	0.000	0.770	1.523	0.727
15	CuAl23	5	3.521	0.000	0.724	1.427	0.677
16	CuAl24	3	1.975	0.444	0.499	0.848	0.437
17	CuAl25	6	3.600	0.111	0.730	1.504	0.686
	Mean	4.41	3.24	0.163	0.685	1.285	0.627
	St. Dev	1.003	0.688	0.176	0.074	0.222	

Note: Na: Observed number of alleles, Ne: Effective number of alleles, Ho: Observed heterozygosity, He: Expected heterozygosity, I: Shannon’s Information index, and PIC: Polymorphic information content.

**Figure 8:**
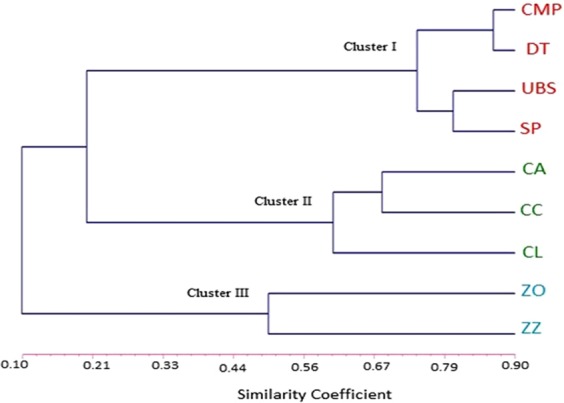
Dendrogram representing the genetic relationship among selected *Curcuma* and *Zingiber*. 17 EST-SSR markers were amplified and analysed for four cultivars of *C*. *alismatifolia* (CMP: Chiang Mai Pink; DT: Doi Tung554; SP: Sweet Pink; UBS: UB Snow 701), three species of *Curcuma* (CL: *C*. *longa*; CC: *C*. *cordata*; CA: *C*. *angustifolia*) and two *Zingiber* species (ZO: *Z*. *officinale*; ZZ: *Z*. *zerumbet*).

Based on Dice’s similarity matrix, we generated a UPGMA hierarchical clustering plot to evaluate the genetic relationships between nine cultivars and species (Fig. 8). The clustering plot clearly grouped the nine cultivars and species within three main clusters. Cluster I included four cultivars of *C*. *alismatifolia* with the highest similarity of 0.73 among its two sub-groups. Cluster II comprised three species of *Curcuma* with less similarity of 0.60 among *C*. *longa* and two *C*. *angustifolia* and *C*. *cordata*, and cluster III consisted of *Z*. *officinale* and *Z*. *zerumbet* with the least similarity of 0.50.

## Discussion

In this study, we report the first analysis of transcriptome data from *C*. *alismatifolia*, a perennial ornamental bulb plant with long vase life and easy growth habits. Some cultivars of this plant have additional medicinal characteristics^41^. In spite of significant advances in DNA/RNA sequencing technologies and progress in developing SSR markers, there is a lack of genetic information to develop molecular markers for assessment of genetic diversity of *C*. *alismatifolia* populations compared to that in other ornamental plant species.

The *C*. *alismatifolia* transcriptome sequence offers a genomic resource for breeding and genetic study of *Curcuma* and provides a reference for plant transcriptome-scale evolutionary analyses of the Zingiberaceae family. *De novo* transcriptome analysis, which is based on RNA-seq data, is a significant alternative method for the whole genome sequencing for organisms with complex and large genomes that comport repetitive sequences^42,43^. In the present study, *de novo* transcriptomes derived from inflorescences of two cultivars of *C*. *alismatifolia* were assembled using Illumina paired-end RNA-seq technology. In total, 13.19 Gb of data was generated after sequencing. N50 and average length of all unigenes was 1,501 bp and 994, respectively (Table 1), which is comparable with N50 reported from the *de novo* transcriptome assembly of *Curcuma longa* (N50 = 1,515 bp)^39^, *Zanthoxylum bungeanum* (N50 = 846 bp)^21^, *Zantedeschia rehmannii* Engl. (N50 = 1476 bp)^44^, *Cicer arietinum* L. (N50 = 1192 bp)^22^, *Euphorbia fischeriana* (N50 = ~1500 bp)^29^, *Cajanus cajan* L. (N50 = 1510 bp)^45^, *Hevea brasiliensis* (N50 = 485 bp)^24^, *Ipomoea batatas* (N50 =  765bp)^26^, and *Camellia sinensis* (N50 = 506 bp)^27^ using the Illumina method. In the assembly analysis, assemblies were measured by the size and accuracy of their transcripts and unigenes. In *de novo* assembly where the reference sequences are not available, assembly evaluation is more difficult. In such cases, some commonly used quality metrics include average length and N50 of unigenes. The unigene N50 is the median unigene size of transcriptome assembly. Although previous studies reported that larger N50 values and longer mean lengths indicate accurate and effective assembly^33,46^, these measures are primitive and often misleading^47^, e.g., N50, one of the most popular reference-free measures, can be maximized by trivial assemblies^48^. The assumption about N50 is that better assemblies will result from a larger number of identified overlaps between input reads and thus will have more reads assembled into longer unigenes. In short, N50 measures the continuity of unigenes but not their accuracy^49^. In transcriptomes, differential N50s for different tissues of the same plant may be observed, since different groups of genes are expressed^50^.

To predict unigene biological functions, unigene functional annotation was performed using six protein databases (Nr, Nt, COG, GO, KEGG, and Swiss-prot, Table 2) producing 48,813 out of 62,105 significant hits (Supplementary Table S1). Employing the same method, the *C*. *alismatifolia* unigene annotation rate was 78.60%, which was higher than that of *C*. *longa* (54.6%). The frequency of unigenes longer than 500 bp within the assembled transcripts may account for a higher percentage of unigene annotation. Generally, the rate of BLAST matches in protein databases for longer unigenes are more likely higher^51^. Other investigators also reported that because the significance of sequence similarity partially depends on the length of the query sequence, higher numbers of short reads in next generation sequencing (NGS) often cannot be matched with known genes^52^. Based on a BLASTX search against the Nr database, 80.5% of annotated unigenes of *C*. *alismatifolia* exhibited similarity to wild banana, *Musa acuminata* ssp. *Malaccensis* ‘DH Pahang’, a perennial monocotyledonous herb of the same order, Zingiberales, as *C*. *alismatifolia* (Fig. 3c). One reason for this similarity could be the absence of whole-genome sequences within public databases created for any species of *Curcuma*. In this study, using the COG database, we classified annotated unigenes into 25 sub-terms or subcategories (Fig. 4), while with the GO database, unigenes were classified into 51 subcategories, including 11, 17, and 23 functions in molecular function, cellular components, and biological process aspects, respectively (Fig. 4), suggesting diverse transcripts in our transcriptome data for *C*. *alismatifolia*^53,54^. These results are comparable with previous studies on *C*. *longa*, calla lily, and *Zanthoxylum bungeanum* showing that unigenes are classified into 25, 24, and 24 subcategories with the GO database and 30, 47, and 43 subcategories with the COG database, respectively^21,39,44^. GO functional annotation helped us describe the macro level of gene functions and predict the physiological role of each unigene^55^. Results illustrated various molecular functions of assembled unigenes, suggesting their involvement in diverse metabolic pathways. Next, 35,629 unigenes were also annotated and mapped to 135 KEGG pathways. To better understand gene interaction and biological function, KEGG pathways were determined to be highly helpful. Functional classification provided based on KEGG pathways revealed that numerous important metabolic pathways within *C*. *alismatifolia* are still unknown and merit further investigation. These findings indicate that *C*. *alismatifolia* makes a huge investment in gene transcription control and capacity, as well as cell maintenance and defence. Approximately 80% of the top 21 hit pathways were involved in genetic information processing and metabolism, whereas the other pathways were related to those involved in cellular process, environmental information processing, human disease, and organismal systems (Fig. 6). In this study, unigenes associated with metabolism of terpenoids and polyketides, cofactors and vitamins, and biosynthesis of other secondary metabolites were also identified, providing evidence that numerous biologically active secondary metabolites have been isolated within *Curcuma* species^41,56–59^. Briefly, functional analysis revealed that RNA-seq-based *de novo* transcriptome analysis for *C*. *alismatifolia*, a non-model organism with a complex genome, will facilitate further research on the physiology, biochemistry, and molecular genetics of *C*. *alismatifolia* or related species.

For gene-based studies detecting functional variations and studying population genetic structure, EST-SSRs are applicable. Transcriptome sequencing, generating enormous amount of sequence data, provides a good resource for development of SSRs. In the present study, out of 62,105 unigenes, 7,811 unigenes containing SSRs, representing approximately 12.57% of the transcriptomic sequences, possess SSR loci, with a distribution density of one SSR locus per 6.6 kb. This rate is comparable with SSR frequencies and distribution density in *Torreya grandis* (2.7%, 25.9 kb)^60^, Pummelo (14.7%, 5.6 kb)^61^, *Amorphophallus* (11.8%, 3.6 kb)^3^, *Zingiber officinale* (2.7%, 25.2 kb)^17^, calla lily (20.34%, 4.1 kb)^44^, *Arachis hypogaea* (17.7%, 3.3 kb)^4^, seagrass (17.5%, 5.8 kb)^62^, three varieties of *Curcuma longa* (14.6%, 5.3 kb; 14.9%, 5.2 kb; 20.5%, 4.8 kb)^39^, and three Macaronesian endemic plant species including *Argyranthemum broussonetii* (2.3%, 27 kb), *Descurainia bourgaeana* (3.5%, 22.9), and *Echium wildpretii* (1.8%, 38.2 kb)^63^. SSR frequency and differences in abundance amongst various species, can be partially attributed to differences between species, the size of the unigene assembly dataset, SSR search criteria, sequence redundancy, and the database-mining tools employed^33,44,64^. In our study, six different repeat motifs were identified, in which the most abundant were trinucleotide repeats (56.35%), followed by dinucleotides (26.44%). In contrast, hexa- (4.74%), penta- (3.35%), and quad- nucleotide repeats (2.61%) were rare (Table 4). This result is similar to previous findings for di- and tri- nucleotide motifs, which are reported as the most frequent SSR motif types within the transcriptome sequences of many other plants, including *C*. *longa*^39^, coloured calla lily^44^, *Amorphophallus* spp.^3^, *Z*. *officinale*^17^, Pummelo^61^, and cotton^65^. Trinucleotide repeats are the most abundant motifs for SSRs because open reading frames do not disturb with insertions and deletions within translated regions, whereas frameshift mutation may restrict the development of other motif types^4,33,66^. Among mononucleotide repeats, as in most plants, A/T repeats were far more abundant than G/C repeats^21,67,68^. The most abundant dinucleotide repeat (20.04%) was the AG/CT motif, as illustrated in Table 4, which is identical to previous findings in *Zanthoxylum bungeanum*^21^ and *Oryza sativa*^69^ followed by AT/TA (4.20%) and AC/GT (2.09%). The most abundant trinucleotide repeat motif in *C*. *alismatifolia* was AGG/CCT (13.82%) and closely followed by CCG/CGG (12.94%), similar to reports in calla lily and *Amorphophallus* spp.^3,17,44^. Consistent with previously reported observations, these results for *C*. *alismatifolia* indicate that the trinucleotide motif CCG/CGG is common in monocots. In addition, we also noticed that GC-rich trinucleotide motifs (CCG/CGG, AGG/CCT, AAG/CTT, AGC/CTG, ACG/CGT, and ACC/GGT > 67%) were more abundant than AT-rich trinucleotide (AAC/GTT, AAG/CTT, AAT/ATT, ACT/AGT, ATC/ATG < 33%). The fact that high GC content and consequent codon usage bias can be considered specific features of monocot genomes is strongly supported by these results^44,70^.

As an efficient way to develop EST-SSR markers, mining transcriptome data provides great flexibility in selecting markers at different resolutions and for different applications^71^. Moreover, SSRs, which are derived from a transcriptome database, have advantages over other strategies, including needing less time and cost. In this study, 17 EST-SSR markers (Table 5) were obtained and verified, offering an informative and applicable approach for evaluation of genetic relationships within *Curcuma* and Z*ingiber* species. Of the 150 primer pairs that were evaluated using a hybrid and three cultivars of *C*. *alismatifolia*, three *Curcuma*, and two *Zingiber* species, 17 polymorphic pairs, as EST-SSR markers, showed a moderate level of genetic diversity (Na = 4.41, Ne = 3.24, Ho = 0.163; He = 0.685, I = 1.285, PIC = 0.627). This can be compared to reports from previous studies on *C*. *longa* (Na = 4.7 and 7.1, PIC = 0.32)^15,72^, Cardamom (Ne = 1.32 to 1.52 and I = 0.22 to 0.36)^73^, and *Aframomum corrorima* (Braun) from the ginger family (Na = 4.82, Ne = 2.09, I = 0.83)^74^ for genomic SSRs. Currently, development of genic SSR markers through NGS-based RNA-seq has become one of the most efficient methods in both model and non-model plants, and many EST-SSR markers have been widely utilized in genetic diversity studies^75,76^. In the present study, the usefulness of the 17 newly developed polymorphic EST-SSR markers for the evaluation of genetic diversity among *Curcuma* and *Zingiber* species was clearly demonstrated. Hierarchical cluster analysis revealed three distinct groups among the nine studied cultivars and species of *Curcuma* and *Zingiber* (Fig. 8), suggesting that the set of cross-species transferable EST-SSR markers developed in this study will enhance the current repository for the genus *Curcuma*. These markers can be useful for detection of markers associated with specific traits in other Zingiberaceae species and related genera in breeding programmes. Cross species transferability has been previously demonstrated among seven species of Zingiberaceae (*Z*. *zerumbet*, *H*. *spicatum*, *C*. *longa*, *C*. *amada*, *C*. *aeruginosa*, *C*. *aromatica and C*. *angustifolia*) for 16 EST-SSR markers from *Z*. *officinale*^17^. In another study, 100% transferability of EST-SSR markers from *C*. *longa* was observed among other species of *Curcuma*^16^. Genic SSRs are more transferable among distantly related species than genomic SSRs because their target coding domains are more likely to be conserved between relatives that makes them valuable for comparative mapping and evolutionary studies^77^.

In addition to the novel markers, the availability of the *C*. *alismatifolia* transcriptome dataset will allow an in-depth exploration of *Curcuma* specific genes with known or unknown biochemical functions along with a better understanding of its economically and agronomically important traits and to develop new cultivars for ornamental purposes or to produce valuable secondary metabolite components.

## Materials and Methods

Rhizomes from C. *alismatifolia* cv. ‘Chiang Mai Pink’ and ‘UB Snow 701’ were obtained from a *Curcuma* Nursery (Ubonrat) in Thailand. Rhizomes were grown in a screen house at field no. 2 within the campus of Universiti Putra Malaysia (UPM), Malaysia. Inflorescences were harvested at the onset of anthesis, instantly frozen in liquid nitrogen, and then stored at −80 °C until RNA extraction.

### RNA extraction, Illumina sequencing and cDNA library construction

Approximately 0.1 g of the upper bracts of two cultivars were frozen, ground in a pre-cooled mortar and pestle using liquid nitrogen, and then transferred into a 2 mL tube. The obtained powder was lysed in 1.5 mL of TRIzol Reagent (Invitrogen, Carlsbad, California, United States) by pipetting up and down. After incubation of the samples for 5 min at room temperature to completely separate nucleoprotein compounds, 0.3 mL of chloroform (one-fifth volume of TRIzol) was added. After shaking firmly for 15 s and incubation at room temperature for 5 min, mixtures were centrifuged at 12,000 g for 5 min at 4 °C to obtain a biphasic solution containing an upper colourless, aqueous phase and a lower phenol-chloroform phase with red colour. The aqueous phase was subsequently transferred into a new Eppendorf tube, and an equal volume of isopropanol was added to precipitate RNA from the aqueous phase. Samples were kept at room temperature for 10 min followed by centrifugation at 18,000 g for 15 min at 4 °C. After discarding the supernatant, 70% ethanol was added to the white RNA pellet and centrifuged at 13,000 g for 5 min at 4 °C. In the final stage, the pellet was air-dried and then dissolved in 30 μl diethyl pyrocarbonate (DEPC)-treated water^78^. Total isolated RNA was treated with DNase I to remove any residual DNA. Using a NanoDrop 2000 (Thermo Fisher Scientific Inc., MA, USA), the purity and concentration of isolated RNA were determined, and quality was verified by electrophoresis on a 1.5% agarose gel.

After total RNA extraction, Oligo (dT) magnetic beads were used to purify and enrich mRNA in each sample. Following purification, a fragmentation buffer was used to break the mRNA. Then, mRNA fragments were used as templates for cDNA synthesis using RT-PCR and random primers. Short cDNA fragments were purified and resolved with elution buffer (EB) for end reparation with T4 DNA and Klenow DNA polymerase. Subsequently, 3′- single adenylation was added to repaired cDNA fragments and connected with sequencing adapters. To construct the cDNA library, these products were purified and amplified via PCR. During quality check steps, Agilent 2100 Bioanaylzer (Agilent Technologies, Santa Clara, CA, USA) and ABI StepOnePlus Real-Time PCR System (Applied Biosystems, Foster City, CA, USA) were used in quantification and qualification of the cDNA library. Finally, cDNA libraries were sequenced on a flow cell using an Illumina HiSeq 4000 system (Illumina, San Diego, CA, USA) with 101-base paired-end reads at BGI Co., Ltd., Shenzhen, China.

### *De novo* assembly and functional annotation of unigenes

After sequencing, raw reads were first filtered for low-quality reads with greater than 20% Q-score <20 bases, adaptor-polluted reads, ambiguous reads containing >5% unknown base (N), and for non-coding RNA such as rRNA, tRNA, and miRNA. After filtering, the remaining reads, called ‘Clean Reads’, were stored in FASTQ format^79^. Clean reads were *de novo* assembled using Trinity (v2.0.6)^80^ software with parameters setting of minimum contig length of 150 bp, min_kmer_cov set to 3 to increase the stringency for reads being assembled together, and sequence homology by 80%. The resulting Trinity sequences were called transcripts, which were obtained from connecting contigs that could not be extended on either end. Then, TGICL (v2.0.6) (TIGR gene indices clustering tool)^81^ was used to cluster transcripts to obtain final unigenes.

Functional annotation of unigenes was performed using BLASTx^82^ against NCBI databases, including Nonredundant (Nr), nucleotide sequence (Nt), Kyoto Encyclopaedia of Genes and Genomes (KEGG)^83^, SwissProt^84^, and Clusters of Orthologous Groups (COG), with an *E*-value threshold of 10^−5^. To obtain the GO annotation, Blast2GO^85^ was applied according to the Nr annotation (GO annotation: http://www.geneontology.org).

### SSR locus detection and primer design

To detect SSRs in unigenes, the Perl script MIcroSAtellite identification tool (MISA, http://pgrc.ipk-gatersleben.de/misa/misa.html)^86,87^ was utilized. To select the SSR loci, the minimum number of repeats was 12 for mono-, six for di-, five for tri- and quad-, four for penta- and hexa- nucleotide repeats, respectively. It was impossible to design primers for SSR loci in which the sequence failed to meet appropriate criteria for designing a primer or whose flanking sequences were too short^21^. SSR primers were designed for each SSR using Primer3 (http://bioinfo.ut.ee/primer3)^88^ according to the following parameters: (1) primer length of 18 to 26 bp with 20 bp as the optimum; (2) PCR product size ranging from 80 to 185 bp; (3) melting temperature (Tm) between 56 °C and 64 °C with a difference of no greater than 4 °C between the Tm values of the forward and reverse primers and with 60 °C as the optimum annealing temperature; (4) GC content of 40% to 70% with an optimum of 50%; (5) designed primer sequence limited to the middle region, with 30 bp being removed from the ends of the contig sequence; and (6) primer pairs devoid of secondary structure or consecutive tracts of a single nucleotide^21^. For this study, 150 total primers were selected at random and synthesized.

### DNA extraction, EST-SSR markers amplification and validation

Total genomic DNA was extracted from young leaves of three *C*. *alismatifolia* cultivars*;* ‘Chiang Mai Pink’, ‘UB Snow 701’, ‘Sweet Pink’, one hybrid ‘Doi Tung 554’, three species of *Curcuma angustifolia*, *Curcuma cordata*, *Curcuma longa*, and two species of *Zingiber officinale* and *Zingiber zerumbet* using a modified extraction buffer of the cetyltrimethyl ammonium bromide (CTAB) protocol^1^. With a NanoDrop ND 2,000 spectrophotometer (Thermo Fisher Scientific Inc., MA, USA), the quality and quantity of DNA were evaluated, and samples were stored at −20 °C before use. Finally, DNA concentration was adjusted to 70 ng/ml.

Amplification and polymorphism of 150 pairs of synthesized SSR primers were tested through polymerase chain reaction (PCR) and electrophoresis. DNA amplification through PCR was performed using a T100 Gradient Thermal cycler (Bio-Rad Laboratories, Inc., CA, USA) within a 15 μL final reaction volume containing 4.5 μL of double distilled water, 1 μL of each primer (100 μmol/L), 1 μL of genomic DNA (70 ng/μL), and 7.5 μL of 2X DreamTaq Green PCR Master Mix (Thermo Fisher Scientific, Inc., MA, USA). PCR condition were as follows: initial denaturation of template DNA at 94 °C for 3 min followed by 35 cycles of 94 °C for 40 s, 50 to 60 °C (depending on the melting temperature of the primer pair used) for 1 min, and 72 °C for 1 min followed by a final extension of 10 min at 72 °C. PCR products were separated on 2.5% MetaPhor™ Agarose gel stained with Midori Green Advance (Nippon Genetics Inc., Japan) using a 50 bp DNA ladder (N3231S, Biolabs, Inc., UK). Gels were visualized under ultraviolet light and imaged using the Gel Doc XR + Imaging system (Bio-Rad Laboratories Inc., CA, USA).

### SSR data analysis

For SSR analysis, PCR products were manually scored based on allele size following data scoring as “0” in the absence of the band and “1” as its presence. The binary data matrix was incorporated in the Numerical Taxonomy and Multivariate Analysis System (NTSYSpc, version 2.10e; Applied Biostatistics Inc. WLB, CAS)^89^ to generate Dice’s similarity matrix. Similarity matrix was used to create a hierarchical clustering plot based on the unweighted paired group method with arithmetic mean (UPGMA) using NTSYSpc software. To calculate genetic diversity parameters, including observed (Ho) and expected heterozygosity (He), and observed (n_a_) and effective number of alleles (n_e_)_,_ Shannon’s Information Index (I) and polymorphic information content (PIC) of selected SSR primers using population genetic analysis software (POPGENE32, ver. 1.32) was employed^90^.

## Supplementary information

Supplement Dataset 1

Supplement Dataset 2

Supplement Dataset 3

## Data Availability

Transcriptome datasets supporting the conclusions of this article are available in the *European Nucleotide Archive (*ENA) under the accession number PRJEB18956.
