# Author Correction: *De novo* assembly of transcriptomes, mining, and development of novel EST-SSR markers in *Curcuma alismatifolia* (Zingiberaceae family) through Illumina sequencing

**DOI:** 10.1038/s41598-019-53129-x

**Published:** 2019-11-05

**Authors:** Sima Taheri, Thohirah Lee Abdullah, M. Y. Rafii, Jennifer Ann Harikrishna, Stefaan P. O. Werbrouck, Chee How Teo, Mahbod Sahebi, Parisa Azizi

**Affiliations:** 1 Department of Crop Science, Faculty of Agriculture, Universiti Putra Malaysia, 43400 Serdang, Selangor Malaysia; 2 Laboratory of Climate-Smart Food Crop Production, Institute of Tropical Agriculture and Food Security, Universiti Putra Malaysia, 43400 Serdang, Selangor Malaysia; 3 Institute of Biological Sciences, Faculty of Science, University of Malaya, 50603 Kuala Lumpur, Malaysia; 4 Centre of Research in Biotechnology for Agriculture (CEBAR), University of Malaya, 50603 Kuala Lumpur, Malaysia; 5 Laboratory of Applied Science In Vitro Plant Biotechnology, Department of Plants and Crops, Faculty of Bioscience Engineering, University Ghent, Valentin Vaerwyckweg 1, BE-9000 Gent, Belgium

Correction to: *Scientific Reports* 10.1038/s41598-019-39944-2, published online 28 February 2019

This Article contains errors in the Author Contributions section.

“All authors had substantial contributions to the conception, design and drafting of this work as individual experts in their fields. In particular, authors S.T. and T.L.A. contributed to organizing the contents of the article and draft writing. Authors M.Y.R., J.A.H., S.P.O.W. and C.H.T. revised the manuscript critically. Authors M.S. and P.A. contributed to the writing, figure preparation and formatting of the article”.

should read:

“Authors S.T., T.L.A., M.Y.R., S.P.O.W., M.S., and P.A. contributed in the conception and design of this work. In particular, authors S.T. and T.L.A. contributed to organizing the contents of the article and writing the draft of the manuscript. Authors M.Y.R., J.A.H., S.P.O.W. and C.H.T. revised the manuscript critically. Authors M.S. and P.A. contributed in writing the draft of the manuscript, figure preparation and formatting of the article”.

